# How do 24-h movement behaviours change during and after vacation? A cohort study

**DOI:** 10.1186/s12966-023-01416-2

**Published:** 2023-03-01

**Authors:** Ty Ferguson, Rachel Curtis, Francois Fraysse, Timothy Olds, Dorothea Dumuid, Wendy Brown, Adrian Esterman, Carol Maher

**Affiliations:** 1 Alliance for Research in Exercise, Nutrition and Activity (ARENA) of the University of South Australia, Adelaide, SA Australia; 2 School of Human Movement and Nutrition Sciences of the University of Queensland, Brisbane, QLD Australia

**Keywords:** Physical activity, Sedentary behaviour, Sleep, Vacation, Holiday, Movement behaviours, 24-h day, Time use

## Abstract

**Background:**

For adults, vacations represent a break from daily responsibilities of work – offering the opportunity to re-distribute time between sleep, sedentary behaviour, light physical activity (LPA) and moderate-to-vigorous physical activity (MVPA) across the 24-h day. To date, there has been minimal research into how activity behaviour patterns change on vacation, and whether any changes linger after the vacation. This study examined how daily movement behaviours change from before, to during and after vacations, and whether these varied based on the type of vacation and vacation duration.

**Methods:**

Data collected during the Annual Rhythms In Adults’ lifestyle and health (ARIA) study were used. 308 adults (mean age 40.4 years, SD 5.6) wore Fitbit Charge 3 fitness trackers 24 h a day for 13 months. Minute-by-minute movement behaviour data were aggregated into daily totals. Multi-level mixed-effects linear regressions were used to compare movement behaviours during and post-vacation (4 weeks) to pre-vacation levels (14 days), and to examine the associations with vacation type and duration.

**Results:**

Participants took an average of 2.6 (SD = 1.7) vacations of 12 (SD = 14) days’ (*N* = 9778 days) duration. The most common vacation type was outdoor recreation (35%) followed by family/social events (31%), rest (17%) and non-leisure (17%).

Daily sleep, LPA and MVPA all increased (+ 21 min [95% CI = 19,24] *p* < 0.001, + 3 min [95% CI = 0.4,5] *p* < 0.02, and + 5 min [95% CI = 3,6] *p* < 0.001 respectively) and sedentary behaviour decreased (-29 min [95% CI = -32,-25] *p* < 0.001) during vacation. Post-vacation, sleep remained elevated for two weeks; MVPA returned to pre-vacation levels; and LPA and sedentary behaviour over-corrected, with LPA significantly lower for 4 weeks, and sedentary behaviour significantly higher for one week. The largest changes were seen for “rest” and “outdoor” vacations. The magnitude of changes was smallest for short vacations (< 3 days).

**Conclusions:**

Vacations are associated with favourable changes in daily movement behaviours. These data provide preliminary evidence of the health benefits of vacations.

**Trial registration:**

The study was prospectively registered on the Australian New Zealand Clinical Trial Registry (Trial ID: ACTRN12619001430123).

**Supplementary Information:**

The online version contains supplementary material available at 10.1186/s12966-023-01416-2.

## Background

The demands of paid and unpaid work substantially reduce a person’s ability to freely allocate their time. A standard workday accounts for approximately half an adult’s waking hours [1, 2]. Additional time may need to be allocated to pre- and post-work related tasks (i.e. dressing, packing meals), along with commuting between home and the workplace. The average daily commute for workers globally is around an hour [3–5]. Further time allocation is needed for essential non-work tasks such as performing domestic duties, caring for loved ones, and attending appointments. Lastly, time must be given to self-care and sleep needs. After accounting for all these daily tasks, little is left to be allocated freely to such things as leisure pursuits, exercise, or rest.

When considering movement behaviours, throughout any day a person’s activities may be considered on a movement continuum where they move between three broad behaviours of sleep, sedentary behaviour, and physical activity [6]. Further, a change in the time spent in one behaviour during a 24-h period necessitates an equal and opposite change across the remaining behaviours [7]. A person’s occupation largely dictates what movement behaviour a person can engage in (e.g. desk-based occupations generally require long periods of sedentary time, while labour-intense occupations require long periods of physical activity). How a person commutes to employment also has an influence on movement behaviours; for example, passive transport (such as public transport) is typically sedentary, whereas active transport (i.e. walking or cycling) requires a degree of physical activity. Finally, the volume and timing of work and essential non-work tasks primarily restrict the window available for sleep, and limits the opportunity for leisure-time sedentary behaviour and physical activity.

Vacations are an extended and relatively uninterrupted break from the usual demands on a person’s time, offering greater freedom in time allocation. Across a calendar year, a person is likely to take several vacations for multiple different purposes [8]. The purpose of vacations varies widely and is specific to the needs of the individual, their income and household demographics [8]. A vacation may occur at home or away, be primarily for leisure (e.g. attending events, visiting attractions), social (visiting family or friends), or active (e.g. hiking, bushwalking) pursuits, for rest and relaxation, or for non-leisure tasks (i.e. caring for others, home renovations).

Health-focused vacation research has largely been directed towards physical and mental health and well-being changes associated with taking vacations. Broadly, vacations are associated with positive changes in self-reported health and well-being [9–11]. Conversely, vacations are also associated with less desirable changes such as overconsumption of food [12]. Less is known about the associations between vacations and movement behaviours. It is generally understood that too little or too much sleep, too little physical activity, and too much sedentary behaviour are associated with poorer health, increased risk of chronic illness, and increased mortality [13–16]. Understanding the association between vacations and movement behaviours offers potential for targeted and timely interventions to improve or maintain favourable levels of each behaviour.

To our knowledge, just two studies have explored adults’ movement behaviours during vacations. De Bloom et al. [17] recruited paid workers in The Netherlands (*n* = 54) who went on vacations of longer than two weeks. Participants self-reported 7.4 h of sleep during vacation compared to 6.7 h pre-vacation. Cooper et al. [18] explored self-reported physical activity and sedentary behaviour levels before, during and after vacation in US adults (*n* = 122). They found a non-significant trend towards an increase in total physical activity during vacation compared to pre-vacation and a significant decrease 6-weeks post-vacation compared to during vacation. Moderate physical activity was significantly lower on vacation compared to pre- and post-vacation whilst vigorous physical activity did not change across time points [18]. In this same study, additional unpublished data were obtained from the authors which showed a non-significant trend towards less sedentary behaviour during vacation compared to pre- and post-vacation [18, 19].

At present, no studies have used objective measures to explore changes in movement behaviours across the 24-h day during vacations. This study aims to address this gap by:

- Describing the characteristics of vacations across a 13-month period (number, duration, timing, key activities)
- Examining how objectively measured daily movement behaviours change from before, to during, and post-vacation periods, and how these vary depending on the type of vacation and vacation duration.
- Identifying the sociodemographic and occupation characteristics associated with favourable changes in movement behaviours during and after vacations.

## Methods

### Study design

This study used data from the *Annual Rhythms In Adults’ lifestyle and health* (ARIA) prospective cohort study which collected daily 24-h movement behaviours in a cohort of Australian adults over a 13-month period. Full details of the study protocol have been previously published [20].

### Setting and procedure

ARIA included a community-based convenience sample of 375 adults, aged 18 to 65 years, recruited from the greater metropolitan area of Adelaide, South Australia.

Participants attended a face-to-face home visit, at which baseline measures (height, weight, and a demographic, health, and lifestyle characteristics survey) were collected and participants were provided with a Fitbit Charge 3 fitness tracker and Fitbit Aria body weight scale (Aria 2 or Aria Air scale; Fitbit Inc., San Francisco, CA, USA). Enrolment in the study occurred in two waves, with wave 1 data collection commencing on 1st Dec 2019 and concluding on 31st Dec 2020, and wave 2 data collection commencing on 1st Dec 2020 and concluding on 31st Dec 2021.

During the study, participants used the Fitbit devices daily and completed eight follow-up surveys which were spread throughout the 13-months, specifically December of the first calendar year, then January, March, April, June, August, October, and December of the second calendar year. These surveys comprised items relating to recreational physical activity, dietary intake, psychological wellbeing, weight perception/management, and work/holiday status. No further face-to-face sessions were needed unless for technical support. Upon completion of the study, participants received an honorarium of $100 and could keep their Fitbit Charge 3 and body weight scale if they wished.

### Participants

Participants were recruited in two ways; either, they were parents of children enrolled in a separate cohort study, call Life on Holidays [21], or they were adults recruited from the general public who had primary (i.e. elementary) school-aged children. Participants from the general public were recruited via advertising in digital media (i.e. Facebook posts and paid advertisements) and print media.

Inclusion criteria were being 18 to 65 years old, being a parent/guardian of a child enrolled in Life on Holidays study or a parent/guardian of a child aged 5 to 12 years, residing in greater metropolitan Adelaide, having access to a Bluetooth-enabled mobile device or computer and home internet, able to understand English, and being ambulant. Exclusion criteria were pregnancy, having an implanted electronic medical device, or experiencing or receiving treatment for any life-threatening condition which impacted daily lifestyle and health.

### Variables

#### Movement behaviours

Objectively measured physical activity, sedentary behaviour and sleep were collected using a wrist-worn Fitbit Charge 3 activity tracker (Fitbit Inc., San Francisco, USA). Participants wore the device on their non-dominant wrist, 24 h a day (except during showering, water-based activities or whilst charging the device) and were asked to sync data to their Fitbit user account at least every 5 days. Data were collected remotely via Fitnesslink software (Portal Australia, Adelaide, Australia), purpose-built for the ARIA study. Minute-by-minute activity data were recorded as sleep, sedentary behaviour, light physical activity, moderate physical activity, vigorous physical activity or non-wear according to Fitbit’s proprietary algorithm. Previous models of Fitbit devices have demonstrated acceptable validity for moderate-to-vigorous physical activity (Flex compared to Actigraph GT3X + *r* = 0.73 [22]), sleep (compared to polysomnography: Charge 2 sensitivity = 0.96, specificity = 0.61; [23] Flex *r* = 0.97 [24]), sedentary time (compared to activPAL: ICC = 0.94, 95% CI: 0.92–0.96 [25]) and total daily energy expenditure (compared to doubly labelled water in free-living conditions: Flex *r*_s_ = 0.84 [26]).

#### Vacation

As part of each follow-up survey, participants reported any dates they had been on vacation since the last survey, if they went away from home on any of those vacation dates. Participants were asked to describe the main purpose of the vacation in an open-ended item. Main purpose responses were grouped into four categories: 1) *Family or social events* (e.g. visiting family, friends, attractions, or attending entertainment events, observing religious or cultural periods (e.g. Christmas, Easter), 2) *Rest and relaxation,* 3) *Outdoor recreation* (e.g. hiking, sports, fishing, boating, camping) or 4) *Non-leisure* (e.g. caring for others, household jobs, medical leave).

#### Employment status

At baseline, participants reported their occupation, whether they worked shift work, and whether they regularly worked on weekends. During each follow-up survey, participants reported their average hours of worked per week, categorised as either no hours, < 15 h, 15–35 h, or ≥ 36 h.

#### Demographics

At baseline, participants’ demographic and health data were collected, including sex, date of birth, marital status, number of children in household, highest education level (below year 10, year 11, year 12 or equivalent, certificate III/IV, advanced diploma/diploma, bachelor’s degree, postgraduate or higher degree), combined gross household income (AUD < $50,000; $50,000-$99,999; $100,000-$199,999; ≥ $200,000) and presence of chronic conditions.

#### Baseline anthropometry

At the baseline home visit, a research assistant measured height (Leicester Height Measure MKII, Invicta Plastics Ltd, Leicester, England) and weight (Fitbit Aria smart scales, Fitbit Inc., San Francisco, USA).

### Statistical analysis

Daily minute totals were calculated for sleep, sedentary behaviour, light physical activity and moderate-to-vigorous physical activity. For a day to be valid, a total of at least 18 h of data and a sleep period was required. Participants were included if they reported at least one vacation and had valid days of movement behaviour data pre-, during, and post-vacation.

For each vacation, mean daily sleep, sedentary, light physical activity, and moderate-to-vigorous physical activity time was calculated for the pre-vacation, during vacation, and post-vacation periods. Pre-vacation was defined as the fourteen days immediately prior to the vacation, excluding any vacation days from a previous vacation. Post-vacation was defined as the 28 days immediately after the vacation, considered in seven-day blocks (i.e. first, second, third and fourth week post-vacation), excluding any vacation days from a subsequent vacation.

All vacation and post-vacation periods were compared to pre-vacation periods for each movement behaviour using multi-level mixed-effects linear regression analyses. Analyses were completed in Stata 17 (StataCorp, College Station, TX, USA) with statistical significance set at 0.05. Random intercepts were used to account for the nested structure of the data (i.e. repeated measures within individuals, individuals within families, and families within waves). Movement behaviours were the dependent variables and were analysed in separate models. The vacation period (i.e. pre-, during or post vacation) was included as a fixed effect. The regression coefficients for vacation were used to identify the difference in daily minutes of movement behaviours during and post-vacation as relative to pre-vacation.

Additional sub-group analyses were completed for type of vacation (i.e., social, rest, outdoor recreation, non-leisure) and length of vacation (i.e., 3 days or less, 4–7 days, 8–14 days, over 14 days) using the same statistical approach as for overall vacation comparisons with each sub-group category analysed in separate models.

Interaction effects of participants’ sociodemographic and occupation characteristics on movement behaviours during vacation were tested using multi-level mixed-effects linear regression (one model per movement behaviour, all sociodemographic and occupational characteristics included in each model). For these models only, Bonferroni corrections (*p*-value multiplied by total variables in the individual model) were used to adjust for the volume of comparisons.

## Results

### Participants

Of the 375 participants recruited, 308 went on at least one vacation where movement behaviour data were available. Participants were predominantly middle-aged (mean age 40.4 years, SD 5.6), overweight or obese, cohabitated, had 2–3 children at home, and were well-educated. Participants who reported paid employment were mostly day-workers (i.e. no shifts or weekends) and performed at least 15 h of work per week (see Table 1). This sample was similar to middle-aged Australian adults in terms of BMI (69% overweight or obese vs 66% of 35- to 44-year-old Australian adults [27]), sex (55.2% women vs 50.8% of 35- to 44-year-old Australian adults [28]) and smoking status (8.4% vs 11.7% of 35- to 44-year-old Australian adults [29]). However, there were slightly more people born in Australia (78.2% vs 67% of 30- to 49-year-old Australian adults [30]), in a domestic relationship (87.7% married or defacto vs 74% of 34- to 44-year-old Australian adults [30]), parents (100% were parents whereas 77% of Australian women aged 30 to 49 years report having children [30]), and employed persons (88.4% reported working vs 75% of 35- to 45-year-old Australian adults [30]). The sample had slightly higher rates of non-school education (83.4% with a non-school qualification vs approximately 75% of 30- to 50-year-old Australian adults [31]).

**Table 1:** Participant and vacation characteristics (*n* = 308)

	**N**	**(%)**	**Mean**	**(SD)**
**Participant characteristics**				
**Age**			40.4	(5.6)
**Weight (kg)**			83.7	(21.2)
**Height (cm)**			170.6	(9.6)
**Female**	170	(55.2)		
**Male**	138	(44.8)		
**Body Mass Index (kg/m**^**2**^**)**				
Underweight	1	(0.3)		
Normal	94	(30.5)		
Overweight	104	(33.8)		
Obese	109	(35.4)		
**Smoker**	26	(8.4)		
**Born in Australia**	241	(78.2)		
**Aboriginal and Torres Strait Islander peoples**	2	(0.6)		
**Marital status**				
Never married	15	(4.9)		
Married/de facto	270	(87.7)		
Separated, divorced or widowed	23	(7.5)		
**Chronic illness**				
None	140	(45.5)		
Single	88	(28.6)		
Multiple	80	(26.0)		
**Adults in household**				
One	28	(9.1)		
Two	264	(85.7)		
Three	12	(3.9)		
Four or more	4	(1.3)		
**Children in household**				
One	27	(8.8)		
Two	171	(55.5)		
Three	79	(25.7)		
Four or more	31	(10.1)		
**Education**				
Year 10 or less	15	(4.9)		
Year 11—12	36	(11.7)		
Certificate/Diploma	107	(34.7)		
University degree	150	(48.7)		
**Occupation**				
Managerial and professional	135	(43.8)		
Technical and clerical	70	(22.7)		
Community, personal service and sales	56	(18.2)		
Machinery operators, drivers, and labourers	12	(3.9)		
No job/other	35	(11.4)		
**Income (Australian dollars)**				
< $50,000	22	(7.1)		
Between $50,000 and $99,999	93	(30.2)		
Between $100,000 and $199,999	151	(49.0)		
≥ $200,000	42	(13.6)		
**Hours worked per week**				
None	31	(10.1)		
< 15	20	(6.5)		
15–35	107	(34.7)		
36 +	150	(48.7)		
**Typical work pattern**				
Weekdays only, no shift	214	(69.5)		
Weekdays and weekends	35	(11.4)		
Weekdays and shifts (inc. nights)	6	(1.9)		
Weekdays, weekends and shifts (inc. nights)	33	(10.7)		
Not applicable	20	(6.5)		
**Vacation characteristics**				
**Total vacations**	806			
**Total vacation days**	9778			
**Vacations per participant**			2.6	1.7
**Vacation duration (days)**			12.1	14.1
**Vacation type (days)**				
Outdoor recreation	2140	35.2		
Family or social events	3140	30.6		
Rest and relaxation	1618	17.1		
Non-leisure activities	2892	16.6		

### The characteristics of vacations

**Fig. 1:**
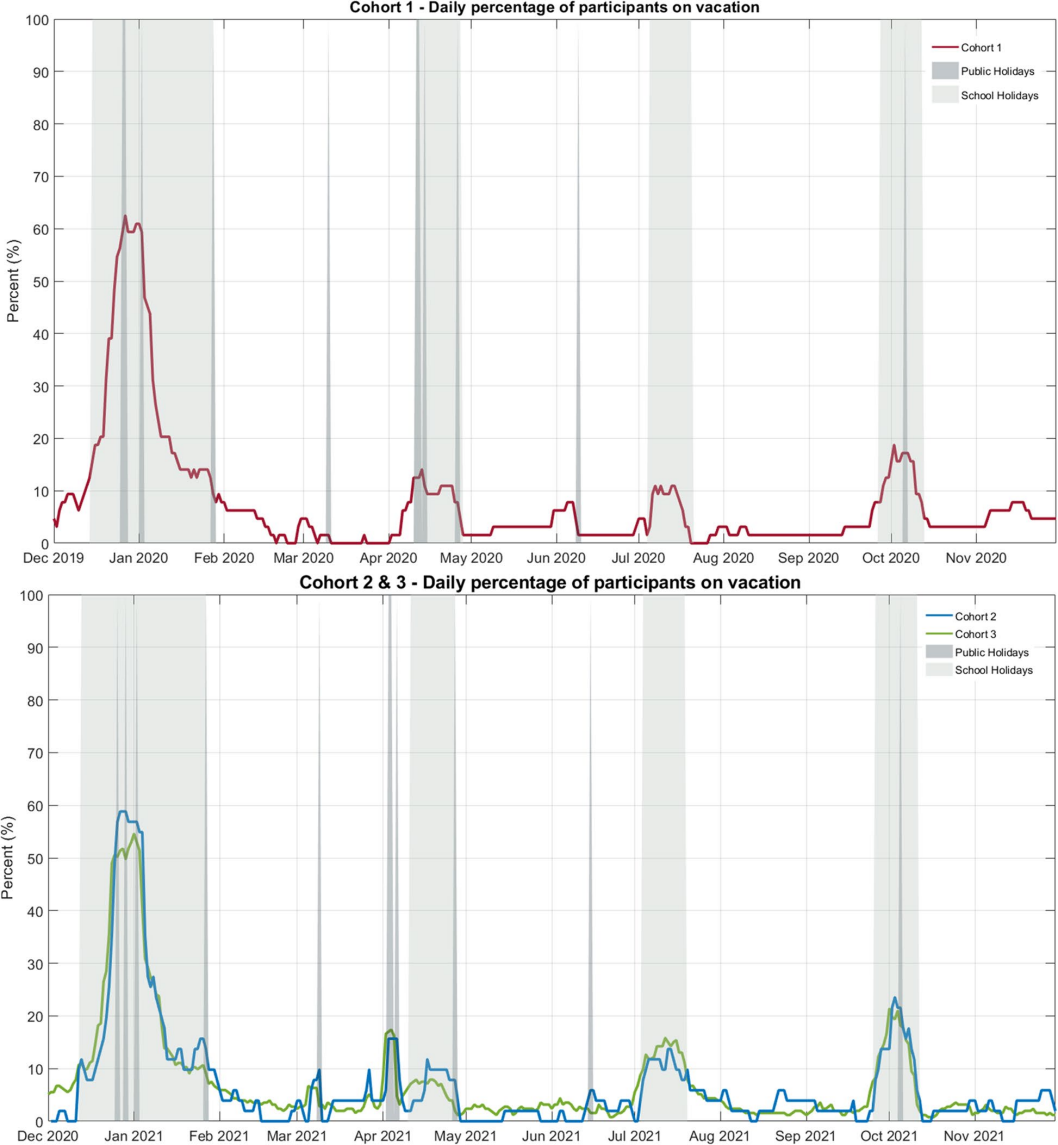
Daily percentage of participants on vacation

A total of 806 vacations were reported. Participants, on average, took less than three vacations per year (mean = 2.6, SD = 1.7) for a duration of 12 days on each occasion (mean = 12.1 days, SD = 14.1 days). The most common vacation purpose reported was *outdoor recreation* (35.2%), followed by *family or social events* (30.6%), *rest and relaxation* (17.1%) and *non-leisure activities* (16.6%). The distribution of vacations across the study period is shown in Fig. 1, showing that participants tended to take vacations more frequently during the Christmas and New Year period (> 50% of participants per day), school holidays, and on public holidays. Activity tracker non-wear time was consistent across the vacation period, with daily averages of 3.8% for pre-vacation, 3.9% during vacation and 3.7% post vacation. Compared to pre-vacation weekdays and weekends, movement behaviours during vacation appear closer to weekend patterns than weekday (Supplementary Fig. 1). Vacation days closely resembled weekend days, with mean differences between vacations and non-vacation weekends ranging from –3.0% for sedentary behaviour to + 2.7% for sleep. By contrast, mean differences between non-vacation weekdays and vacations were very wide, ranging from –14.8% for MVPA to + 8.1% for sedentary behaviour.

### Changes in movement behaviours across the vacation period

**Fig. 2:**
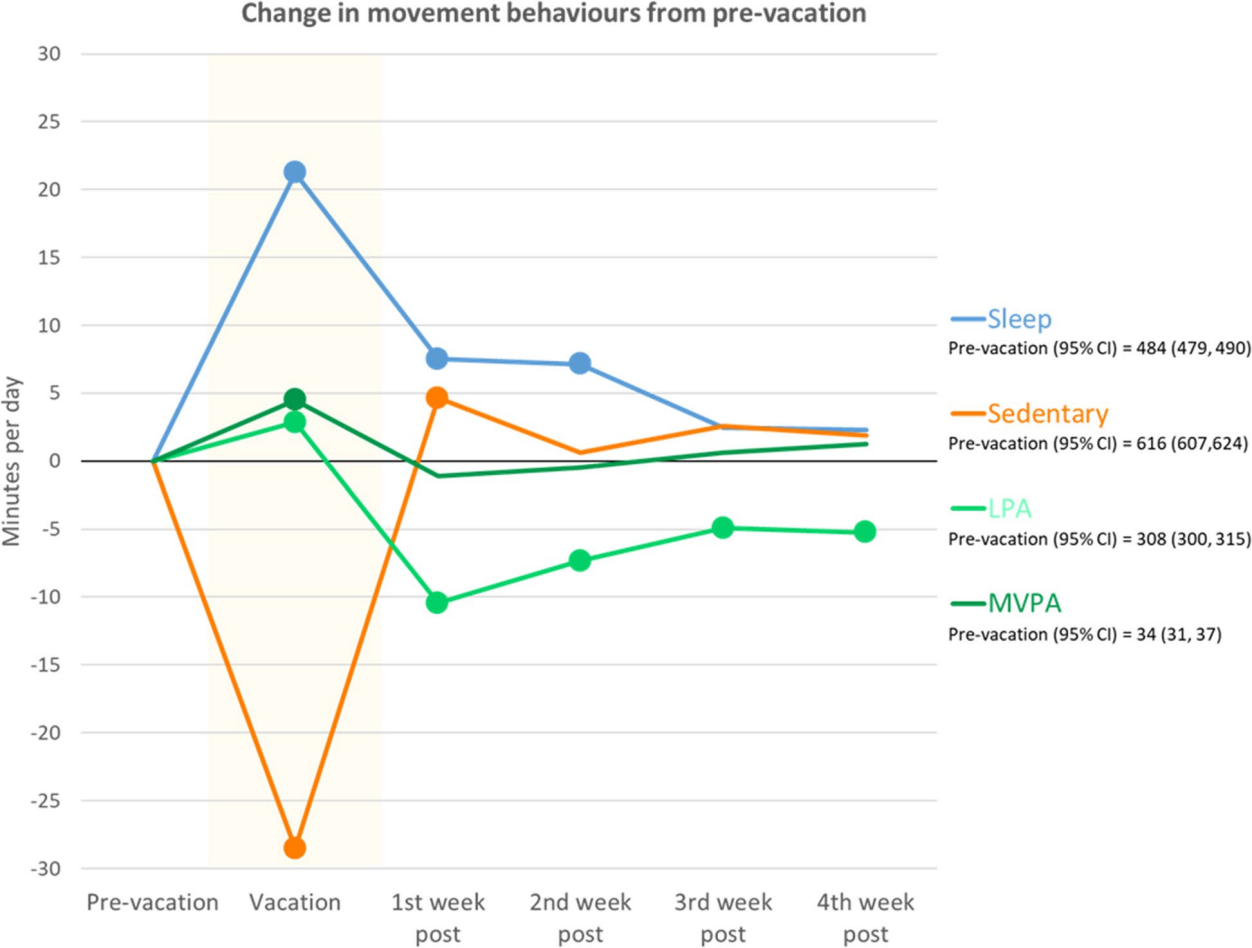
Change in movement behaviours from pre-vacation. Figure notes: • = significant change from pre-vacation (*p *< 0.05). Pre-vacation is calculated as the mean of the 14 days immediately prior to any vacation. Vacation includes any vacation reported (mean length = 12.1 days, standard deviation = 14.1 days). LPA = light physical activity, MVPA = moderate-to-vigorous physical activity. Values reported in Supplementary Table 1

The average daily minutes spent in sleep, sedentary behaviour, and light, moderate and vigorous physical activity, over the vacation period are shown in Fig. 2.

Sleep increased significantly during vacation compared with pre-vacation (+ 21 min/day [95% CI =  + 19, + 24], p < 0.001, + 4.4% change relative to pre-vacation). This significant increase persisted into the first and second weeks post-vacation (+ 8 min/day [95% CI =  + 4, + 11], *p* < 0.001, + 1.5% and + 7 min/day [95% CI =  + 4, + 10], *p* < 0.001, + 1.5% respectively) before returning to near-baseline levels.

Sedentary behaviour decreased significantly during vacation compared with pre-vacation (-29 min/day [95% CI = -32, -25], *p* < 0.001, -4.6%). In contrast, post-vacation sedentary behaviour increased significantly from pre-vacation levels (+ 5 min/day [95% CI =  + 1, + 9], *p* = 0.022, + 0.7%).

During and post-vacation time points were all significantly different to pre-vacation levels of light physical activity, however the direction differed. Light physical activity increased during vacation (+ 3 min/day [95% CI = 0, + 5], *p* = 0.021, + 0.9%) before decreasing relative to pre-vacation levels and persisting across each post-vacation timepoint (first week = -10 min/day [95% CI = -13,-8] *p* < 0.001, -3.4%; second week = -7 min/day [95% CI = -10, -5] *p* < 0.001, -2.4%; third week = -5 min/day [95% CI = -8, -2] *p* = 0.001, -1.6%; fourth week = -5 min/day [95% CI = -8, -2] *p* < 0.001, -1.7%).

A significant increase in moderate-to-vigorous physical activity occurred during vacation (+ 5 min/day [95% CI =  + 3, + 6], p < 0.001, + 13.2%) before returning to pre-vacation levels immediately afterwards.

### Vacation characteristics associated with changes in movement behaviours

#### Main vacation purpose

**Fig. 3:**
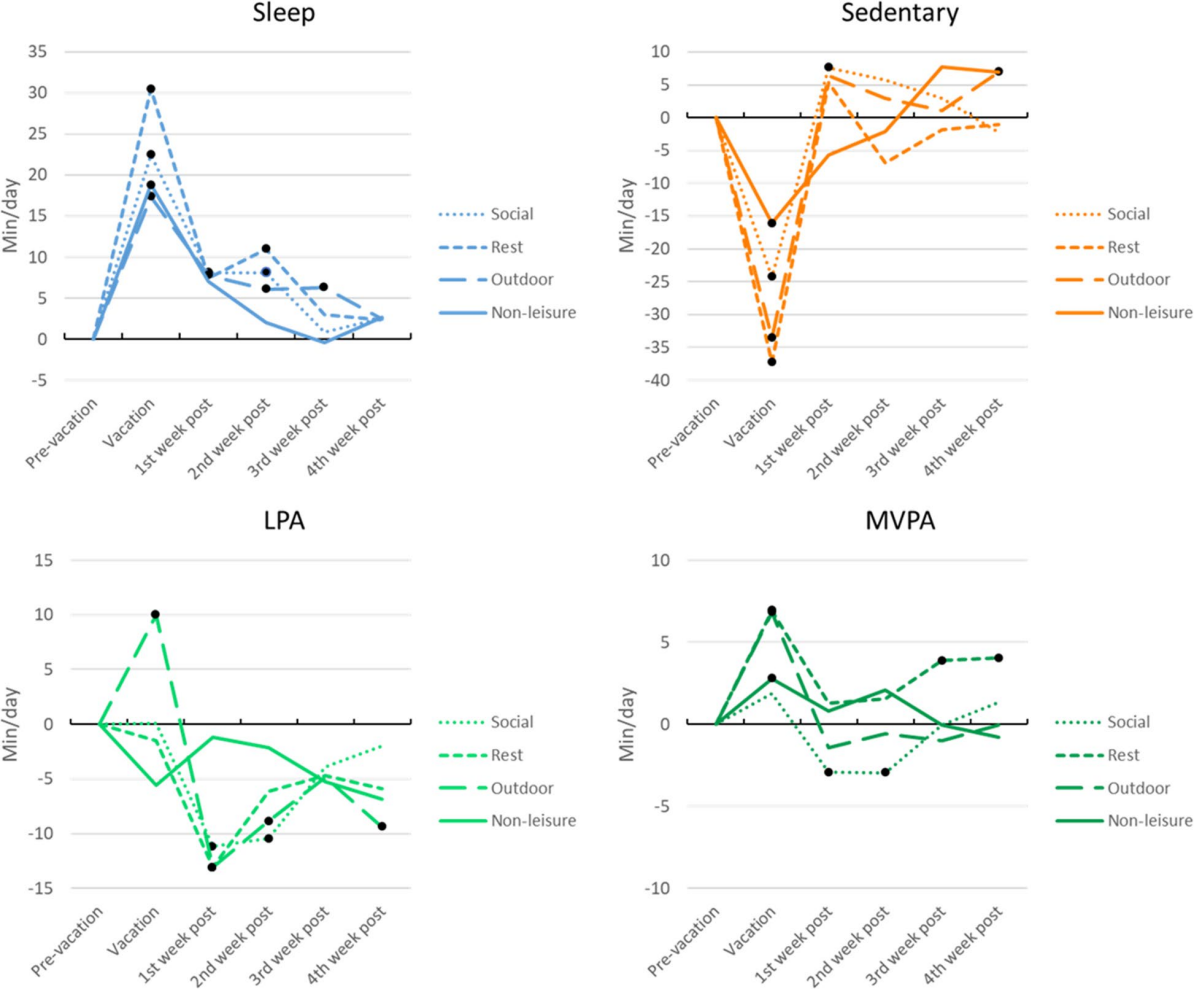
Change in movement behaviours from pre-vacation by type of vacation. Figure note: • = significant change from pre-vacation (*p *< 0.05). LPA = light physical activity, MVPA = moderate-to-vigorous physical activity. Values reported in Supplementary Table 1. Social = Family or social events, e.g. visiting family, friends, attractions, or attending entertainment events, observing religious or cultural periods (Christmas, Easter), Rest = Rest and relaxation, Outdoor = Outdoor recreation, e.g. hiking, sports, fishing, boating, camping, Non-leisure = non-leisure tasks, e.g. caring for others, household jobs, medical leave

Movement behaviours across the vacation period according to main purpose of the vacation (i.e. family/social events, rest, outdoor recreation and non-leisure) were broadly similar to the overall vacation period patterns (Fig. 3 and Supplementary Table 1). Generally, increases in sleep, reductions in sedentary time, minimal changes in light physical activity, and small increases in moderate-to-vigorous physical activity were observed during vacation. Changes post-vacation appear to persist longer after outdoor recreation and family/social vacations than for rest and non-leisure vacations. Over-correction in the first week post-vacation occurred for sedentary behaviour after family/social vacations (decrease during, increase post) and light physical activity after outdoor vacations (increase during, decrease post).

#### Vacation length

**Fig. 4:**
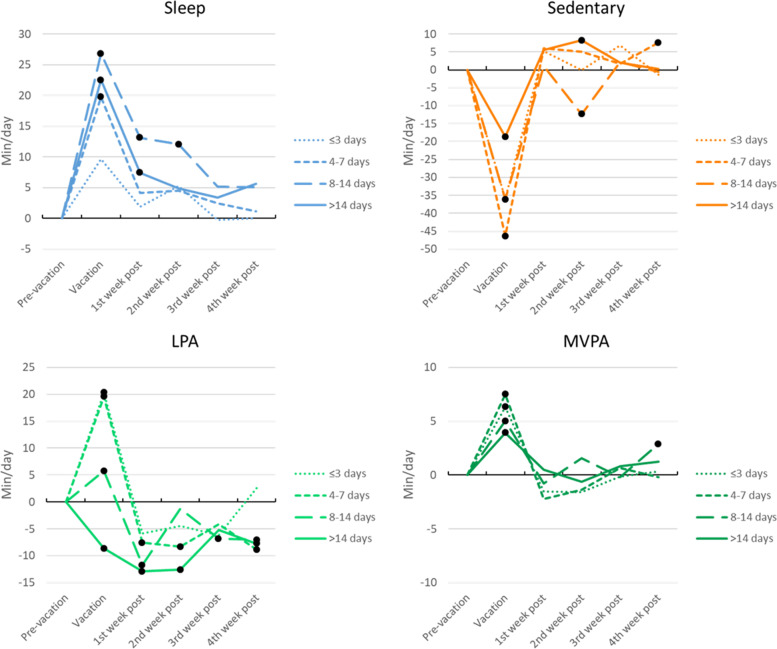
Change in movement behaviours from pre-vacation by length of vacation. Figure note: • = significant change from pre-vacation (*p* < 0.05). LPA = light physical activity, MVPA = moderate-to-vigorous physical activity. Values reported in Supplementary Table 1

Movement behaviour changes over the vacation period appeared to differed by vacation length (summarised in Fig. 4; full details of means, 95% confidence intervals, and *p* values are provided in Supplementary Table 1). Broadly, it appears the longer the vacation, the larger the change in sleep, and the smaller the change in sedentary behaviour, light and moderate-to-vigorous physical activity. Directions of change during vacation were largely consistent with overall patterns (sleep and physical activity increased, sedentary behaviour decreased, see Fig. 2), except for light physical activity during long vacations (> 2 weeks) where it decreased significantly by -9 min/day (95% CI = -12, -5, *p* < 0.001, -2.8%) from pre-vacation levels. In addition, the longevity of changes seems related to vacation length, with behaviours returning to pre-vacation levels almost immediately after short vacations, whereas changes persisted for several weeks after longer vacations for sleep and light physical activity.

### Sociodemographic and occupation characteristics associated with changes in movement behaviours across the vacation period

Supplementary Table 2 contained results of interaction effects of participants’ sociodemographic and occupation characteristics on movement behaviours during vacation. The changes in daily movement behaviours during vacation were unrelated to sociodemographic characteristics such as sex, age, education level, BMI, health status, occupational characteristics, and baseline activity levels. No comparisons were statistically significant post-Bonferroni corrections.

## Discussion

### Principal findings

Across all vacations, participants’ movement behaviour patterns changed significantly in favourable directions (i.e., increased physical activity, decreased sedentary behaviour). The duration of vacation and type of vacation appeared to be associated movement behaviour changes, with vacations of 4 days to 1–2 weeks and outdoor recreation vacations associated with the most favourable changes. The largest significant during vacation changes were seen for vacations of > 1 week duration. Additionally, after vacations of > 1 week duration, sleep remained higher and light physical activity lower than pre-vacation for at least the first week. Finally, participant characteristics did not appear to influence changes in movement behaviours during or after vacation.

This study is the first to explore movement behaviours across the 24-h day using a longitudinal design with objective measures. Our findings were consistent with the two previous studies using self-reported measures. For example, de Bloom et al. [17] reported an increase in vacation sleep duration for vacations of longer than two-weeks. Of note, the change was almost twice as large as the current study (+ 42 min/day vs + 23 min/day), which may be due to the method of measuring sleep. Furthermore, our findings for increased physical activity and decreased sedentary behaviour during vacation were broadly consistent with those reported by Cooper et al. [18], who reported non-significant trends, rather than significant findings as found in the current study. This may be due to the sample size, since the Cooper study involved 122 participants, which was less than half the sample size in our study.

The most consistent favourable changes across movement behaviours occurred in the 4 days to 2-week range, with these changes appearing to persist longer, the longer the vacation. This is consistent with the law of inertia, where people have set patterns of time use which are difficult to perturb [32]. When a perturbation does occur, the tendency is for behaviour to snap back to initial patterns once the short-term disruption (i.e. a vacation) is removed [32]. The longer the disruption, the greater the change, and the slower the return to pre-existing levels [32].

When considering the type of vacation, outdoor recreation was the only category where all movement behaviours had significant favourable changes during vacation. This may be because outdoor recreation vacations are ideally suited to physical activity with two important facilitators, the natural environment and free time, available in abundance [33]. Further, increased physical activity has been shown to have a positive effect on sleep quality, particularly duration [34, 35].

Most participants in this study worked in office-based occupations, on a Monday-to-Friday schedule. The large decrease in sedentary behaviour from weekdays to weekends is consistent with previous literature of office workers [36] and is also observed in the change from pre-vacation to during vacation. The opposite change occurred for sleep and physical activity, with both increasing on weekends and during vacations, relative to weekdays and pre-vacation respectively. The overall consistent effect of weekends and vacations on movement behaviours is likely a product of people not needing to go to work, removing long hours of sedentary employment, and increasing time available to freely allocate across movement behaviours. This would suggest vacations are like very long weekends. When considering post-vacation, the over-correction in sedentary behaviour observed during the first week, where it was significantly increased, may be attributed to a workload catch-up, where tasks accumulated during vacation may need to be added to usual work demands [37].

### Strengths and limitations

To our knowledge, this is largest ever study of movement behaviour patterns during vacations, as well as the first study of vacations that captures 24-h objective movement behaviour data. Use of a remote data collection method meant there were minimal barriers to participants’ involvement in the study when away from home.

This study explored activity behaviour patterns exclusively, with minimal knowledge of participant motivations, vacation destinations, itineraries or vacation companions. This sample had a high concentration of vacations during the Australian summer (December and January) and it is possible the time of year when a vacation is taken may be associated with movement patterns, however, this was beyond the scope of the current study. The study sample was generally representative of middle-aged Australians. However, as the sampling frame was parents of primary school age children, when compared with national population data there were slight over-representation of people who were married / in de facto relationships, parents, employed persons and those with higher education. It must be acknowledged that the participants were all parents and city-dwellers residing in one major Australian city, and that the generalisability of the findings, especially for adults without children (approximately 23% of middle aged Australians) and those who reside in non-urban areas (14% of Australians) is unclear. The statistical analyses used in this study explored the 24-h day using individual models without direct comparison between movement behaviours. An alternative and more complex approach to account for direct interactions would be compositional data analysis. Whilst the sample size is adequately powered for overall analyses, it’s possible sub-group analyses on participant characteristics may be underpowered. Finally, data collection occurred during the COVID-19 pandemic. Importantly, within state travel was minimally impacted in South Australia during the study period. However, interstate and international travel was largely restricted. Australia adopted an elimination approach to the virus with widespread and varied border closures along with strict quarantine rules, adding up to two additional weeks to any cross-border travel. Participant choices of vacation destination, duration and vacation activities are likely to have differed from pre-COVID travel.

### Implications

Whilst the findings of this study suggest there may be an optimal length and type of vacation to yield the greatest favourable changes, the availability of vacation leave will ultimately dictate vacation options. Minimum yearly paid leave entitlements vary widely around the world. In Australia, the minimum is 4 weeks [38], whereas some European countries are higher (e.g. 6 weeks in France [38]). The US has no minimum paid leave requirements [39], though about three-quarters of US workers receive 2–3 weeks of paid leave [40]. Workers in countries with low allocations, such as the US, may prefer multiple short breaks, rather than a single 2-week block each year. Conversely, high allocation countries such as France, allow for multiple 2-week vacations per year, where potentially favourable changes may be repeated and spread across the year. Additionally, leave availability may influence vacation type as some options may not be feasible on short breaks where travel time to the destination is prohibitive.

### Future directions

Changes observed during vacation are likely the result of a combination of factors, specific to each vacation. Whilst it is difficult to replicate this outside of the vacation setting, potential exists for interventions around the vacation. Future research should include experimental study designs, exploring interventions that encourage favourable behaviour change in the lead up to or after vacations. An interesting finding from the current study is the over-correcting of sedentary behaviour and light physical activity post-vacation toward less favourable directions which would suggest interventions aimed at reducing sedentary behaviour and increasing light physical activity at this timepoint may be warranted. There is potential to explore what specific behaviours a person engages with during vacation (i.e., increased walking, earlier bedtime) that likely produced favourable changes and aim to incorporate these habits into daily life post-vacation. Another important consideration is exploring potential changes in mental health and well-being during vacation and how they are associated with movement behaviours. Exploring sleep changes in chronic under-sleepers would be of interest to observe if the same increases in sleep duration observed in this study are replicated in that population. Finally, a larger sample or a sample followed for a longer duration would allow more in-depth explorations into particular sub-groups identified within this study, i.e. vacation purpose, demographic subgroups.

## Conclusion

During vacations, favourable changes in movement behaviours (i.e., more physical activity and less sedentary behaviour) were observed, with these changes most pronounced in vacations of 4 days to 2 weeks long, and during vacation for outdoor recreation. Post-vacation, moderate-to-vigorous physical activity quickly returned to pre-vacation levels whereas sedentary behaviour and light physical activity tended to over-correct. Increases in sleep were apparent for around two weeks post-vacation, particularly after vacations longer than one week, and after outdoor and social vacations. These data provide preliminary evidence of the health benefits of vacations. Findings suggest that interventions timed immediately post-vacation may help combat unfavourable over-corrections observed in sedentary behaviour and light physical activity.

## Supplementary Information

**Additional file 1:** **Supplementary Figure 1.** Pre-vacation weekdays and weekends as a percent difference from vacation days.

**Additional file 2: Supplementary Table 1.** Coefficients, standard errors and *p* values of data presented in Figs. 2, 3 and 4.

**Addtional file 3: Supplementary Table 2.** During vacation changes in movement behaviours by participant characteristics.

## Data Availability

Data used in the current study are available and may be obtained from the corresponding author upon reasonable request.

## Abbreviations

ARIA: Annual Rhythms In Adults’ lifestyle and health study
BMI: Body mass index
LPA: Light physical activity
MVPA: Moderate-to-vigorous physical activity
