# Supplementary material for: How do 24-h movement behaviours change during and after vacation? A cohort study
Source: Int J Behav Nutr Phys Act. 2023 Mar 1;20:24. doi: 10.1186/s12966-023-01416-2 (PMC9976678; doi:10.1186/s12966-023-01416-2)
Supplement: Supplementary file 1 — Additional file 1: Supplementary Figure 1. Pre-vacation weekdays and weekends as a percent difference from vacation days. [file 12966_2023_1416_MOESM1_ESM.pdf]

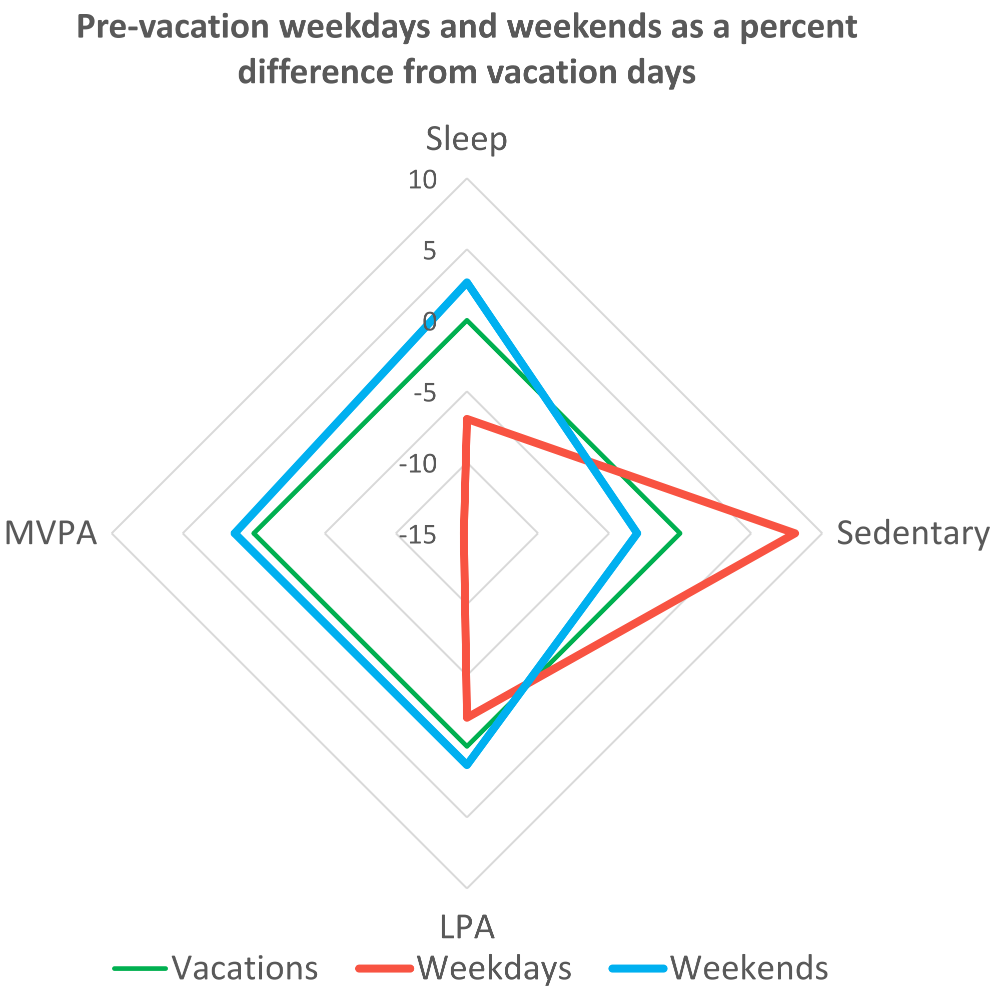

Supplementary Figure 1: Pre-vacation weekdays and weekends as a percent difference from vacation days.

Notes : LPA = light physical activity, MPVA = moderate-to-vigorous physical activity.
