# Supplementary material for: How do 24-h movement behaviours change during and after vacation? A cohort study
Source: Int J Behav Nutr Phys Act. 2023 Mar 1;20:24. doi: 10.1186/s12966-023-01416-2 (PMC9976678; doi:10.1186/s12966-023-01416-2)
Supplement: Supplementary file 2 — Additional file 2: Supplementary Table 1. Coefficients, standard errors and p values of data presented in Figs. 2, 3 and 4. [file 12966_2023_1416_MOESM2_ESM.pdf]

Supplementary Table 1: Coefficients, standard errors and p values of data presented in Figures 2, 3 and 4.

			Change from pre-vacation									
		Pre-vacation	Vacation		1st week post		2nd week post		3rd week post		4th week post	
		cons [95% CI]	coeff [95% CI]	p	coeff [95% CI]	p	coeff [95% CI]	p	coeff [95% CI]	p	coeff [95% CI]	p
Pre, during and post vacation activity profiles (Figure 2)												
All vacations	Sleep	484.3 [478.7, 489.8]	21.2 [18.6, 23.9]	<0.001	7.5 [4.4, 10.6]	<0.001	7.1 [4.0, 10.3]	<0.001	2.5 [-0.7, 5.7]	0.131	2.3 [-1.0, 5.5]	0.172
	Sedentary	615.5 [606.9, 624.2]	-28.5 [-31.9, -25.2]	<0.001	4.6 [0.7, 8.6]	0.022	0.6 [-3.4, 4.7]	0.760	2.6 [-1.5, 6.6]	0.219	1.9 [-2.3, 6.0]	0.377
	LPA	307.6 [300.4, 314.9]	2.8 [0.4, 5.3]	0.021	-10.4 [-13.3, -7.6]	<0.001	-7.3 [-10.2, -4.5]	<0.001	-4.9 [-7.8, -2.0]	0.001	-5.2 [-8.2, -2.3]	<0.001
	MVPA	34.0 [31.3, 36.7]	4.5 [3.4, 5.6]	<0.001	-1.1 [-2.5, 0.2]	0.096	-0.5 [-1.9, 0.8]	0.465	0.6 [-0.7, 2.0]	0.370	1.3 [-0.1, 2.7]	0.072
Vacation activity profiles by type of vacation (Figure 3)												
Social	Sleep	486.1 [477.3, 494.9]	22.5 [17.9, 27.0]	<0.001	8.1 [2.9, 13.4]	0.002	8.1 [2.7, 13.5]	0.003	0.8 [-4.6, 6.2]	0.769	2.5 [-2.9, 8.0]	0.360
	Sedentary	611.1 [598.0, 624.2]	-24.3 [-30.1, -18.5]	<0.001	7.6 [0.9, 14.3]	0.025	5.7 [-1.2, 12.6]	0.104	2.9 [-4.0, 9.8]	0.408	-2.2 [-9.2, 4.7]	0.532
	LPA	309.8 [299.0, 320.5]	0.1 [-4.0, 4.2]	0.953	-11.2 [-15.9, -6.5]	<0.001	-10.5 [-15.3, -5.6]	<0.001	-3.9 [-8.8, 1.0]	0.119	-2.0 [-6.9, 2.9]	0.423
	MVPA	34.2 [30.5, 37.9]	1.9 [-0.1, 3.8]	0.065	-2.9 [-5.2, -0.7]	0.011	-3.0 [-5.3, -0.6]	0.013	-0.1 [-2.4, 2.3]	0.953	1.3 [-1.0, 3.7]	0.265
Rest	Sleep	481.7 [471.8, 491.5]	30.5 [23.9, 37.1]	<0.001	7.4 [-0.1, 15.0]	0.055	11.0 [3.4, 18.6]	0.005	2.9 [-4.7, 10.6]	0.448	2.4 [-5.4, 10.2]	0.547
	Sedentary	627.4 [612.5, 642.2]	-37.3 [-45.4, -29.3]	<0.001	5.3 [-3.9, 14.6]	0.257	-6.9 [-16.3, 2.4]	0.146	-1.8 [-11.2, 7.5]	0.700	-1.1 [-10.6, 8.4]	0.821
	LPA	299.7 [286.7, 312.7]	-1.5 [-7.0, 4.1]	0.603	-13.1 [-19.4, -6.7]	<0.001	-6.2 [-12.6, 0.3]	0.060	-4.7 [-11.1, 1.7]	0.148	-5.9 [-12.4, 0.7]	0.080
	MVPA	33.3 [28.0, 38.6]	6.9 [4.2, 9.6]	<0.001	1.3 [-1.8, 4.4]	0.418	1.5 [-1.6, 4.7]	0.336	3.9 [0.7, 7.0]	0.016	4.0 [0.8, 7.2]	0.014
Outdoor	Sleep	480.7 [473.6, 487.7]	17.4 [12.7, 22.0]	<0.001	7.8 [2.5, 13.0]	0.004	6.1 [0.9, 11.3]	0.023	6.3 [1.0, 11.7]	0.020	2.5 [-2.9, 7.8]	0.370
	Sedentary	613.7 [601.2, 626.3]	-33.6 [-39.6, -27.6]	<0.001	6.4 [-0.4, 13.1]	0.065	2.9 [-3.8, 9.6]	0.395	1.0 [-5.8, 7.9]	0.770	7.0 [0.1, 13.9]	0.048
	LPA	310.9 [300.7, 321.1]	10.0 [5.7, 14.3]	<0.001	-13.1 [-17.9, -8.2]	<0.001	-8.9 [-13.7, -4.0]	<0.001	-4.8 [-9.8, 0.1]	0.057	-9.4 [-14.4, -4.4]	<0.001
	MVPA	36.5 [32.7, 40.3]	6.8 [4.8, 8.8]	<0.001	-1.4 [-3.7, 0.8]	0.216	-0.6 [-2.8, 1.7]	0.627	-1.0 [-3.3, 1.3]	0.392	-0.1 [-2.4, 2.3]	0.955
Non-leisure	Sleep	487.5 [476.8, 498.2]	18.8 [12.3, 25.2]	<0.001	7.1 [-0.7, 14.8]	0.074	2.0 [-5.8, 9.9]	0.614	-0.4 [-8.4, 7.6]	0.921	2.6 [-5.6, 10.9]	0.527
	Sedentary	607.3 [590.4, 624.2]	-16.1 [-24.4, -7.9]	<0.001	-5.7 [-15.6, 4.1]	0.253	-2.1 [-12.1, 7.9]	0.675	7.8 [-2.5, 18.0]	0.137	6.9 [-3.5, 17.4]	0.193
	LPA	313.9 [299.1, 328.7]	-5.5 [-11.5, 0.4]	0.068	-1.2 [-8.2, 5.9]	0.748	-2.1 [-9.3, 5.0]	0.559	-5.3 [-12.6, 2.1]	0.161	-6.8 [-14.4, 0.7]	0.075
	MVPA	32.9 [27.7, 38.1]	2.8 [0.1, 5.5]	0.046	0.8 [-2.4, 4.1]	0.620	2.1 [-1.2, 5.4]	0.214	-0.1 [-3.4, 3.3]	0.969	-0.8 [-4.2, 2.7]	0.658
Vacation activity profiles by length of vacation (Figure 4)												
≤ 3 days	Sleep	481.5 [470.9, 492.1]	9.6 [-1.4, 20.7]	0.087	1.9 [-5.4, 9.2]	0.616	5.3 [-2.2, 12.8]	0.169	-0.3 [-7.9, 7.4]	0.945	0.1 [-7.9, 8.0]	0.988
	Sedentary	615.6 [598.8, 632.4]	-36.0 [-50.3, -21.6]	<0.001	5.2 [-4.3, 14.7]	0.287	-0.1 [-9.9, 9.7]	0.988	6.8 [-3.2, 16.7]	0.182	-1.3 [-11.6, 9.0]	0.806
	LPA	309.3 [294.9, 323.6]	20.3 [10.8, 29.9]	<0.001	-5.8 [-12.2, 0.5]	0.069	-4.4 [-10.9, 2.1]	0.180	-6.2 [-12.8, 0.4]	0.067	2.6 [-4.2, 9.5]	0.452
	MVPA	35.1 [30.1, 40.2]	6.3 [1.7, 10.9]	0.007	-1.5 [-4.5, 1.5]	0.326	-1.6 [-4.7, 1.5]	0.314	-0.2 [-3.3, 3.0]	0.921	0.3 [-2.9, 3.6]	0.840
4 to 7 days	Sleep	486.5 [479.3, 493.8]	19.7 [13.9, 25.6]	<0.001	4.1 [-0.9, 9.2]	0.109	4.5 [-0.6, 9.5]	0.082	2.5 [-2.7, 7.6]	0.347	1.1 [-4.0, 6.3]	0.668
	Sedentary	617.5 [604.7, 630.4]	-46.3 [-54.2, -38.5]	<0.001	6.0 [-0.9, 12.8]	0.087	5.0 [-1.8, 11.8]	0.153	1.6 [-5.3, 8.5]	0.641	7.6 [0.6, 14.5]	0.033
	LPA	304.3 [294.0, 314.6]	19.6 [14.1, 25.1]	<0.001	-7.6 [-12.4, -2.8]	0.002	-8.3 [-13.1, -3.6]	0.001	-4.2 [-9.0, 0.6]	0.089	-8.9 [-13.7, -4.0]	<0.001
	MVPA	33.9 [29.9, 37.9]	7.5 [4.9, 10.1]	<0.001	-2.2 [-4.5, 0.04]	0.054	-1.4 [-3.7, 0.9]	0.236	0.6 [-1.7, 3.0]	0.582	-0.2 [-2.5, 2.1]	0.861
8 to 14 days	Sleep	475.9 [467.6, 484.2]	26.8 [21.1, 32.5]	<0.001	13.1 [6.5, 19.8]	<0.001	12.0 [5.5, 18.6]	<0.001	5.2 [-1.5, 11.9]	0.128	5.1 [-1.7, 11.8]	0.141
	Sedentary	616.4 [603.0, 629.7]	-36.1 [-43.3, -29.0]	<0.001	1.0 [-7.2, 9.3]	0.805	-12.4 [-20.6, -4.2]	0.003	2.1 [-6.3, 10.4]	0.624	-0.5 [-8.9, 7.9]	0.910
	LPA	313.0 [301.4, 324.5]	5.7 [0.7, 10.8]	0.027	-11.8 [-17.6, -5.9]	<0.001	-1.3 [-7.1, 4.5]	0.661	-6.8 [-12.7, -0.9]	0.024	-7.1 [-13.0, -1.1]	0.021
	MVPA	36.4 [31.8, 41.0]	5.0 [2.6, 7.4]	<0.001	-0.8 [-3.6, 2.0]	0.575	1.5 [-1.2, 4.3]	0.276	-0.2 [-3.0, 2.6]	0.900	2.9 [0.03, 5.7]	0.048
> 14 days	Sleep	483.1 [475.2, 490.9]	22.5 [18.4, 26.7]	<0.001	7.4 [1.8, 13.1]	0.010	4.9 [-0.9, 10.7]	0.101	3.4 [-2.4, 9.2]	0.248	5.6 [-0.1, 11.4]	0.054

Sedentary	612.4 [600.4, 624.4]	**-18.7 [-23.8, -13.6]**	**<0.001**	5.5 [-1.5, 12.5]	0.122	**8.2 [1.1, 15.4]**	**0.024**	2.0 [-5.1, 9.1]	0.586	0.2 [-6.8, 7.3]	0.945
LPA	311.2 [301.4, 321.1]	**-8.6 [-12.4, -4.8]**	**<0.001**	**-12.9 [-18.1, -7.8]**	**<0.001**	**-12.6 [-17.8, -7.3]**	**<0.001**	-5.2 [-10.4, 0.1]	0.053	**-7.8 [-13.0, -2.5]**	**0.004**
MVPA	34.3 [30.7, 37.9]	**3.9 [2.2, 5.7]**	**<0.001**	0.5 [-1.8, 2.9]	0.671	-0.6 [-3.0, 1.8]	0.602	0.8 [-1.6, 3.2]	0.503	1.2 [-1.1, 3.6]	0.309

Notes: Results of multi-level mixed-effects linear regression analyses shown. One model per movement behaviour, i.e. for "all vacations" results (Figure 2), four models were used. Bold values denotes significant change from pre-vacation (p<0.05). Coeff = coefficient, cons = constant, CI = confidence interval, LPA = light physical activity, MVPA = moderate-to-vigorous physical activity. No Bonferroni corrections were applied to the analyses reported in this Table.
