# Supplementary material for: How do 24-h movement behaviours change during and after vacation? A cohort study
Source: Int J Behav Nutr Phys Act. 2023 Mar 1;20:24. doi: 10.1186/s12966-023-01416-2 (PMC9976678; doi:10.1186/s12966-023-01416-2)
Supplement: Supplementary file 3 — Addtional file 3: Supplementary Table 2. During vacation changes in movement behaviours by participant characteristics. [file 12966_2023_1416_MOESM3_ESM.pdf]

Supplementary Table 2: During vacation changes in movement behaviours by participant characteristics.

		Sleep			Sedentary			LPA			MVPA		
		Coeff	[95% CI]	p	Coeff	[95% CI]	p	Coeff	[95% CI]	p	Coeff	[95% CI]	p
Vacation change (all characteristics = cons)		30.4	[-9.0, 69.7]	0.130	-41.8	[-104.2, 20.6]	0.189	12.5	[-38.4, 63.4]	0.630	3.1	[-13.8, 19.9]	0.721
Sex	cons = male												
	female	-4.2	[-16.1, 7.6]	0.482	11.9	[-7.2, 30.9]	0.222	-9.5	[-25.2, 6.1]	0.234	-3.0	[-8.0, 2.1]	0.251
Age		-0.1	[-0.9, 0.7]	0.824	0.2	[-1.2, 1.5]	0.801	0.0	[-1.1, 1.1]	0.977	0.0	[-0.3, 0.4]	0.899
Education	cons = high school												
	certificate/diploma	-3.0	[-17.7, 11.6]	0.685	-10.0	[-33.1, 13.1]	0.397	12.9	[-5.8, 31.6]	0.177	4.3	[-2.1, 10.6]	0.189
	university degree	-1.9	[-16.9, 13.0]	0.799	-15.0	[-38.6, 8.7]	0.215	16.7	[-2.6, 36.0]	0.089	2.6	[-3.9, 9.0]	0.438
Body Mass Index	cons = normal/under												
	overweight	-14.6	[-25.7, -3.6]	0.009	17.1	[-0.7, 34.8]	0.060	-2.1	[-16.8, 12.5]	0.775	-1.7	[-6.4, 3.0]	0.476
	obese	-4.4	[-15.6, 6.9]	0.448	11.8	[-6.2, 29.8]	0.199	-8.6	[-23.4, 6.2]	0.255	-0.7	[-5.5, 4.1]	0.775
Chronic conditions	cons = none												
	one or more	-0.9	[-5.2, 3.4]	0.692	2.3	[-4.5, 9.1]	0.513	-2.2	[-7.8, 3.3]	0.432	0.2	[-1.7, 2.0]	0.864
National physical activity guidelines	cons = below												
	meets	-4.3	[-18.3, 9.6]	0.542	-12.1	[-34.3, 10.1]	0.285	4.4	[-13.6, 22.5]	0.630	4.9	[-1.1, 10.9]	0.108
	exceeds	-7.6	[-21.8, 6.5]	0.290	-8.3	[-30.8, 14.2]	0.470	7.6	[-10.7, 25.9]	0.414	-0.5	[-6.6, 5.5]	0.864
Occupation	cons = none												
	Machinery operators, drivers and labourers	1.4	[-34.3, 37.0]	0.941	19.2	[-37.9, 76.4]	0.510	-26.5	[-73.6, 20.6]	0.271	1.4	[-13.4, 16.1]	0.856
	Community, personal service and sales	-3.6	[-31.5, 24.2]	0.800	34.0	[-11.8, 79.8]	0.146	-29.8	[-68.0, 8.5]	0.127	-1.9	[-13.2, 9.4]	0.740
	Technical and clerical	0.8	[-26.1, 27.8]	0.953	4.6	[-39.8, 49.0]	0.840	2.4	[-34.7, 39.6]	0.898	0.4	[-10.4, 11.3]	0.942
	Managerial and professional	-4.9	[-30.9, 21.0]	0.709	-4.8	[-47.9, 38.4]	0.828	16.5	[-19.9, 52.9]	0.375	0.5	[-9.7, 10.8]	0.917
Shift worker	cons = no												
	yes	-14.3	[-32.3, 3.8]	0.121	21.7	[-6.8, 50.2]	0.136	8.1	[-15.0, 31.2]	0.492	-2.0	[-9.8, 5.8]	0.617
Weekend worker	cons = no												
	yes	3.8	[-10.0, 17.6]	0.588	-15.0	[-36.9, 7.0]	0.182	2.7	[-15.3, 20.7]	0.768	2.0	[-4.0, 7.9]	0.511
Work hours per week	cons = none												
	<15hr	6.8	[-23.9, 37.4]	0.666	2.4	[-47.8, 52.5]	0.926	-3.3	[-45.1, 38.5]	0.876	-1.4	[-14.0, 11.1]	0.821
	≥15 to <36hr	20.1	[-7.5, 47.7]	0.153	-1.9	[-48.0, 44.2]	0.935	-21.9	[-60.8, 17.0]	0.271	-2.4	[-13.2, 8.4]	0.664
	≥36hr (full-time)	16.5	[-10.0, 43.0]	0.223	-9.2	[-53.9, 35.4]	0.685	-15.2	[-53.1, 22.7]	0.433	2.2	[-8.1, 12.4]	0.679

Notes: Results of multi-level mixed-effects linear regression analyses shown. One model per movement behaviour (column). All changes relative to pre-vacation (14-days immediately prior to vacation). Coeff = coefficient (relative to characteristic cons value), cons = constant (comparator / y-intercept), CI = confidence interval, LPA = light physical activity, MVPA = moderate-to-vigorous physical activity. P-values presented above are pre-Bonferroni correction. After correction, no p-values were significant ($p<0.05$).
